# Four Inducible Promoters for Controlled Gene Expression in the Oleaginous Yeast *Rhodotorula toruloides*

**DOI:** 10.3389/fmicb.2016.01666

**Published:** 2016-10-21

**Authors:** Alexander M. B. Johns, John Love, Stephen J. Aves

**Affiliations:** Department of Biosciences, University of ExeterExeter, UK

**Keywords:** *Rhodotorula toruloides*, inducible promoters, *NAR1*, *ICL1*, *CTR3*, *MET16*, in-yeast assembly, G418 resistance

## Abstract

*Rhodotorula* (*Rhodosporidium*) *toruloides* is an oleaginous yeast with great biotechnological potential, capable of accumulating lipid up to 70% of its dry biomass, and of carotenoid biosynthesis. However, few molecular genetic tools are available for manipulation of this basidiomycete yeast and its high genomic GC content can make routine cloning difficult. We have developed plasmid vectors for transformation of *R. toruloides* which include elements for *Saccharomyces cerevisiae* in-yeast assembly; this method is robust to the assembly of GC-rich DNA and of large plasmids. Using such vectors we screened for controllable promoters, and identified inducible promoters from the genes *NAR1, ICL1, CTR3*, and *MET16*. These four promoters have independent induction/repression conditions and exhibit different levels and rates of induction in *R. toruloides*, making them appropriate for controllable transgene expression in different experimental situations. Nested deletions were used to identify regulatory regions in the four promoters, and to delimit the minimal inducible promoters, which are as small as 200 bp for the *NAR1* promoter. The *NAR1* promoter shows very tight regulation under repressed conditions as determined both by an EGFP reporter gene and by conditional rescue of a *leu2* mutant. These new tools facilitate molecular genetic manipulation and controllable gene expression in *R. toruloides*.

## Introduction

*Rhodotorula toruloides* (formerly *Rhodosporidium toruloides*; Wang et al., 2015) is a red, oleaginous, basidiomycete yeast. It can accumulate lipid up to 70% of its dry biomass and metabolize a variety of low cost carbon sources, which makes it of interest for biofuel production (Kosa and Ragauskas, 2011; Wiebe et al., 2012). Other proposed uses of this organism include production of carotenoids or other high value chemicals (Lee et al., 2014), as a biocontrol agent (Buck and Andrews, 1999) and as a source of phenylalanine ammonia-lyase for treatment of phenylketonuria (Gilbert and Tully, 1982).

Although *R. toruloides* has great biotechnological potential, as a basidiomycete it is distantly related to more commonly employed yeast such as *Saccharomyces cerevisiae*. Also, *R. toruloides* has a high genomic GC content (62%; Kumar et al., 2012) and consequently a strong bias in its codon usage, necessitating codon optimization of protein coding transgenes (Liu et al., 2013). As a result, molecular genetic tools developed for other fungi are rarely directly transferable to *R. toruloides.* In order to perform routine molecular genetic manipulations in this organism, a new toolset must be developed.

Inducible promoters are required for controllable expression of heterologous genes in *R. toruloides*. Several constitutive promoters have been isolated from *R. toruloides* and used to express transgenes, including promoters from the genes *GPD1, FBA1, PGK1, PGI1* and *TPI1* (Liu et al., 2013; Wang et al., 2016). There is, however, a paucity of inducible promoters characterized for use in *R. toruloides*. The recently isolated *DAO1* promoter is strongly induced when D-amino acids are provided as a carbon source, however, it cannot be completely repressed which is a disadvantage for expression of proteins which impede cell growth (Liu et al., 2015). In addition the D-amino acids required for induction are expensive and may be prohibitive for large, industrial fermentations. Finally, induction or repression conditions may affect the results of an experiment. Therefore it would be beneficial to have more than one regulatable promoter available for use in *R. toruloides*.

In this study we extend the range of molecular tools for expression of genes in the oleaginous yeast *R. toruloides*. A system based on in-yeast assembly has been designed for construction of plasmids for transformation of *R. toruloides* using selectable markers including G418 resistance. Potential inducible promoters were screened using an EGFP reporter and we identify and characterize a toolset of four controllable promoters with different induction/repression conditions for use in *R. toruloides*.

## Materials and Methods

### Strains and Media

*Rhodotorula toruloides* wild-type haploid strain CBS 14 (*MAT-A1*; ATCC 10788, IFO 0559, MTCC 457; (Rennerfelt, 1937)) and NCYC 1585 (*MAT-A2 leu2*^-^ *ino*; (Tully, 1985)), were obtained from the Centraalbureau voor Schimmelcultures, Utrecht, The Netherlands and The National Collection of Yeast Cultures, Norwich, UK, respectively. NCYC 1585 is a mutant derivative of wild-type haploid strain CBS 349 (*MAT-A2*; ATCC 10657, IFO 0880). *R. toruloides* was grown at 30°C in YPD (Sambrook and Russell, 2001), or Yeast Nitrogen Base without amino acids (YNB; ForMedium, Hunstanton, UK) with 20 g L^-1^ glucose. Promoter induction and repression media were YNB with 20 g L^-1^ glucose modified as follows: for *SGA1* induction medium, glucose was replaced with maltose; for *ICL1* and *ICL2* induction medium glucose was replaced with 200 mM sodium acetate; for *NAR1* induction medium, YNB without ammonium sulfate was supplemented with 0.78 g L^-1^ potassium nitrate; for *THI5* and *THI4* induction medium YNB without thiamine was used and for repression medium 20 mg L^-1^ thiamine was included; for *MET16*, 1 mM methionine was included in repression medium; for *CCC2* induction medium contained 20 μM CuSO_4_ and repression medium was formulated without copper; for *CTR3* and *CTR31* initial screens induction medium was formulated without copper and repression medium contained 20 μM CuSO_4_, for time course and promoter cut-down experiments induction medium contained 100 μM bathocuproinedisulfonic acid (BCS). Solid media contained 2% agar except for *NAR1* induction/repression media where 2% agarose was used.

*Agrobacterium tumefaciens* strain GV3101 (van Larebeke et al., 1974) was grown at 28°C in LB (Cold Spring Harbor, 2006) containing rifampicin (50 μg mL^-1^). Cloning was performed using *Escherichia coli* NEB5α (New England Biolabs, Ipswich, MA, usa) grown in LB at 37°C. In-yeast assembly was performed using *S. cerevisiae* strain BY4742 (*MATα his3*Δ *leu2*Δ *lys2*Δ *ura3*Δ; Brachman et al., 1998) grown in YPD, or YNB with 20 g L^-1^ glucose and Complete Supplement Mix without uracil (ForMedium, Hunstanton, UK) for auxotrophic selection.

### General Molecular Biological Techniques

Chemicals were supplied by Sigma-Aldrich (St Louis, MO, USA) unless indicated. Synthetic DNA was produced by GeneArt (ThermoFisher, Waltham, MA, USA). DNA manipulation was performed using standard techniques (Sambrook and Russell, 2001). PCR was performed using Q5 polymerase (New England Biolabs, Ipswich, MA, USA) with oligonucleotides purchased from Eurofins (Ebersberg, DE). Plasmid DNA was prepared from *E. coli* by alkaline lysis using a GeneJET Plasmid Miniprep Kit (ThermoFisher, Waltham, MA, USA). Restriction digests were performed using High-Fidelity restriction endonucleases (New England Biolabs, Ipswich, MA, USA). All cloning was verified by Sanger sequencing (Source Biosciences, Nottingham, UK).

*E. coli* was chemically transformed using High Efficiency Transformation (New England Biolabs, Ipswich, MA, USA). Chemically competent *A. tumefaciens* was prepared and transformed by the protocol of Holsters et al. (1978).

### *S. cerevisiae* In-yeast Assembly

In-yeast assembly was performed by a modified version of the protocol of Kilaru and Steinberg (2015). Briefly, DNA fragments with overlapping homology regions of 25 bp at their ends were co-transformed into *S. cerevisiae* in an approximately equimolar ratio using a yeast transformation kit (Sigma-Aldrich, St Louis, MO, USA). In-yeast assembled plasmids were extracted by the method of Singh and Weil (2002), transformed into *E. coli*, isolated by alkaline lysis and verified by Sanger sequencing of junctions.

### Plasmid Construction

Plasmid pG418-Rt was constructed using Gibson assembly (Gibson et al., 2009). pCAMBIA0380 (Cambia, Canberra, ACT, Australia) was digested using *Pvu*I; the *R. toruloides* CBS 14 *GPD1* promoter was amplified from genomic DNA using primers RtGPD1F-pCambia0380 and GPD1R-G418 (**Table 1**). These two components were assembled with a codon-optimized APH(3′) G418 resistance gene amplified using primers G418F-GPD1 and G418R-pCambia0380, using an NEB Gibson Assembly Cloning Kit (New England Biolabs, Ipswich, MA, USA).

**Table 1:** Primers used.

Primer name	Sequence^1^
RtGPD1F-pCambia0380	cacgtgtgaattacaggtgaccagctcgaatttccccgatCTGCAGAACTACGCCCTCTC
GPD1R-G418	tgcgtcttctccttgcccatTGTGAGTGATCTGGTGTTGTTC
G418F-GPD1	acaacaccagatcactcacaATGGGCAAGGAGAAGACGCA
G418R-pCambia0380	ttcaatcttaagaaactttattgccaaatgtttgaacgatcgCTAGAAGAACTCGTCGAGCATGAG
RtGPD1F-pCambia0380-2	ggcgcgccgaattcgagctcggtacccaaCTGCAGAACTACGCCCTCGC
G418-NcTerm	cagaggagcctgaatgttgagtggaatgatCTAGAAGAACTCGTCGAGCA
LeuF-pCambia0380	ctcacccgtccaactcccaccctcccacgtgcagcccaccATGCCCTACTCTATCACCTGCTTG
LeuR-NcTerm	ctactcacacattattatggagaaaactagtTCACTTCTTGGTAAGCAATCCCGT
ICL1-1500-F	gaccggcaacaggattcaatGTTCTACAAGGACGTTTGGC
ICL1-800-F	gaccggcaacaggattcaatGTCCTGCGCAGCGGCG
ICL1-600-F	gaccggcaacaggattcaatTGGTGCGTTCGCGTGCGT
ICL1-400-F	gaccggcaacaggattcaatGGACCGCATCCCGTGCGTC
ICL1-200-F	gaccggcaacaggattcaatACTTTGACTCGCATTACACTTTTTTCTCCGC
ICL1-100-F	gaccggcaacaggattcaatGGCTTTCTTTCTCTCTCTGCGAACGAGG
ICL1-R	ttcgagaccggatccgccatCTCGTGTGTAGTGTCGT
ICL2-1500-F	gaccggcaacaggattcaatCGCCGGCCGACCACCACTA
ICL2-R	ttcgagaccggatccgccatGGCGTGCACTCGTGACA
SGA1-1500-F	gaccggcaacaggattcaatCTCGGCAAGCACAGCTTGATG
SGA1R	ttcgagaccggatccgccatCGTGAGCGGGAGAGCG
NAR1-1500-F	gaccggcaacaggattcaatTGCGTCCGTCTCTCGGT
NAR1-800-F	gaccggcaacaggattcaatGTCTCCGCAGAATCGTCGGACC
NAR1-600-F	gaccggcaacaggattcaatAGCAGCTCTCGTCTTGTCGCTTGG
NAR1-400-F	gaccggcaacaggattcaatCAACGTCGGCCCGCCTTGT
NAR1-200-F	gaccggcaacaggattcaatCGGACAGCAACTCTGGCTCTGG
NAR1-100-F	gaccggcaacaggattcaatCGCTGGTCTTGTTGGACAGCTGG
NAR1-R	ttcgagaccggatccgccatTCTGCTAGTGCTGTAGGTG
THI5-1500-F	gaccggcaacaggattcaatTGCGTCCGTCTCTCGGT
THI5-R	ttcgagaccggatccgccatTCTGCTAGTGCTGTAGGTG
THI4-1500-F	gaccggcaacaggattcaatGCAGAGCAAGAAGAACC
THI4-R	ttcgagaccggatccgccatGTTGATTCTTAAACGTC
MET16-1500-F	gaccggcaacaggattcaatGCAAGGTGTTGGAGATGTC
MET16-800-F	gaccggcaacaggattcaatATAGAGCGCCATCTTCTCGAGC-
MET16-600-F	gaccggcaacaggattcaatAGGCGGGCTGCTGAAGG
MET16-400-F	gaccggcaacaggattcaatCGGGCGTCGCAGGC
MET16-200-F	gaccggcaacaggattcaatCTGTGTGCGCCCGACTTG
MET16-100-F	gaccggcaacaggattcaatCGCGTGCTTCGCTCTTG
MET16-R	ttcgagaccggatccgccatCTGTTGAGGGTGCG
CCC2-1500-F	gaccggcaacaggattcaatCAGCGGAGTCTGTCGGTCGA
CCC2-R	ttcgagaccggatccgccatGGCGAACTCGGGCGA
CTR3-1500-F	gaccggcaacaggattcaatAGGTACTTGGAGAGGGCTGC
CTR3-800-F	gaccggcaacaggattcaatGGGCACGCGGAGGG
CTR3-600-F	gaccggcaacaggattcaatCGCAAAAACAGCGCATCC
CTR3-400-F	gaccggcaacaggattcaatTCTCCCAGCCGCTCCTCTAG
CTR3-200-F	gaccggcaacaggattcaatTGGGGTCGCTCTGAGGG
CTR3-100-F	gaccggcaacaggattcaatGCACGCAGCCTCAACCG
CTR3-R	ttcgagaccggatccgccatCGCGGATCGCAGAT
CTR31-1500-F	gaccggcaacaggattcaatGCGCAACGCACGGAGACC
CTR31-R	ttcgagaccggatccgccatCGTTCAGCAAGCGCACG
Icl1R-Leu	ccaagcaggtgatagagtagggcatCTCGTGTGTAGTGTCGT
Nar1R-Leu	ccaagcaggtgatagagtagggcatGTTCGTGGGTCGTTCTTC
Met16R-Leu	ccaagcaggtgatagagtagggcatCTGTTGAGGGTGCG
Ctr3R-Leu	ccaagcaggtgatagagtagggcatCGCGGATCGCAGAT

*^1^Priming sequences are shown in uppercase and 5′ extensions in lowercase.*

pEGFP-Rt-YR-G418 was constructed in two steps by in-yeast assembly of plasmid pC-G418-YR (Sidhu et al., 2015) digested with *Pvu*II, and codon-optimized G418 resistance gene under regulation of the *GPD1* promoter amplified from pG418-Rt using primers RtGPD1F-pCambia0380-2 and G418-NcTerm; the resulting plasmid was digested using *Hin*dIII and assembled in-yeast with synthetic DNA comprising the *R. toruloides PGK1* promoter, codon-optimized EGFP gene, and CMV35S terminator.

Plasmids for testing promoter activity were constructed by in-yeast assembly of *Afl*II/*Pml*I-digested pEGFP-Rt-YR-G418 with promoter fragments amplified from genomic DNA using respective primers (**Table 1**).

Plasmid pLeu-Rt-YR-G418 was constructed by amplification of the *R. toruloides* CBS 14 *LEU2* gene using primers LeuF-pCambia0380 and LeuR-NcTerm, and in-yeast assembly with pEGFP-Rt-YR-G418 digested with *Pml*I and *Spe*I. Plasmids for conditional *leu2*^-^ rescue under the regulation of *ICL1, NAR1, MET16* and *CTR3* 1500-bp promoter fragments were assembled in the same manner as plasmids for testing promoter activity with EGFP, with the modifications that pLeu-Rt-YR-G418 was used instead of pEGFP-Rt-YR-G418 as the base plasmid, and reverse primers Icl1R-Leu, Nar1R-Leu, Met16R-Leu and Ctr3R-Leu were used for amplification of *ICL1, NAR1, MET16* and *CTR3* promoters, respectively.

### Transformation of *R. toruloides*

Transformation of *R. toruloides* was performed using a modified version of the protocol of Liu et al. (2013). *A. tumefaciens* containing the appropriate binary plasmid was grown in LB with rifampicin (50 μg mL^-1^) and kanamycin (50 μg mL^-1^) at 28°C for 48 h, then diluted to an OD of approximately 0.1 in induction medium (Gelvin, 2006) at 24°C for 6 h. A 200 μL volume of this *A. tumefaciens* culture was then mixed with 200 μL of an overnight culture of *R. toruloides*, spread over a nitrocellulose membrane on solid induction medium and incubated at 24°C for 48 h. Membranes were transferred to YPD with G418 (150 μg mL^-1^) and cefotaxime (150 μg mL^-1^) and incubated at 30°C for 2–3 days. Colonies were restreaked to fresh selective YPD and grown overnight.

### Measurement of EGFP Expression

For initial screening and promoter cut-down experiments three independent transformants were each grown overnight in YNB, pelleted by centrifugation (2500 × *g* for 5 min) and washed twice with sterile water. Approximately 10^7^ cells were added to 20 mL induction/repression medium and allowed to grow for 16 h (8 h for *MET16* promoter cut-down experiments). Samples of 0.5 ml were then taken and kept on ice until fluorescence could be measured. For measurement of induction rates, starter cultures were grown overnight in repressive conditions. Cells were then pelleted by centrifugation and washed twice with sterile water. Approximately 10^7^ cells were added to 50 mL induction or repression medium and grown at 30°C. Samples of 0.5 mL were taken at the indicated time intervals and kept on ice until fluorescence could be measured.

Fluorescence was quantified by flow cytometry using a FACSAria II (BD Biosciences, San Jose, CA, USA) with excitation at 488 nm and a 530/30 nm emission filter. To quantify cell density, CountBright absolute counting beads (ThermoFisher, Waltham, MA, USA) were added to samples. Data were analyzed using FlowJo software (FlowJo, Ashland, OR, USA) to determine median fluorescence for each sample. Student’s *t*-tests were conducted to determine statistical significance between different experimental conditions.

### Auxotrophic Rescue

Cells were grown overnight in induction or repression medium with leucine (100 mg L^-1^) as indicated, harvested by centrifugation, washed twice, then re-suspended in sterile water to approximately 10^6^ cells mL^-1^. A 10x serial dilution was then spotted on to solid induction or repression media with or without leucine using a replica plater (Sigma-Aldrich, St Louis, MO, USA).

### Motif Discovery

Motif discovery was performed using MEME, version 4.11.2 hosted at http://meme-suite.org/tools/meme using default settings. Genomes used for comparison were: *R. toruloides* CBS 14 (Kumar et al., 2012), *R. toruloides* CBS 349 (Zhang et al., 2016), *R. graminis* WP1 (Firrincieli et al., 2015), *Sporobolomyces* (formerly *Sporidiobolus*) *salmonicolor* CBS 6832 (Coelho et al., 2015), *S. roseus* JGIBAIF-5F1, *Phyllozyma* (formerly *Sporobolomyces*) *linderae* CBS 7893, *Microbotryum lychnidis-dioicae* p1A1 (Grigoriev et al., 2014), *Mixia osmundae* IAM 14324 (Toome et al., 2014), *Leucosporidium creatinivorum* (formerly *Leucosporidiella creatinivora*; Grigoriev et al., 2014) and *Puccinia graminis* (Duplessis et al., 2011). Searching for known elements within promoters was performed using FIMO, version 4.11.2 hosted http://meme-suite.org/tools/fimo using default settings.

## Results and Discussion

### Identification of Candidate Inducible Promoters in *R. toruloides*

To identify a toolset of inducible promoters for use in different situations, we screened potential inducible promoters based on successful use in other fungi. Orthologs of promoters regulated by carbon source, nitrogen source, metabolite availability, and copper availability were identified in the *R. toruloides* CBS 14 haploid genome by reciprocal BLASTP hits against their respective genes, and are listed in **Table 2**. This work focused on the *R. toruloides* haploid strain CBS 14 as its lipid production is well characterized (Evans and Ratledge, 1984; Wiebe et al., 2012, Zhang et al., 2016), the genome has been sequenced (Kumar et al., 2012; Zhang et al., 2016), and it is almost identical to strain NP 11 (Zhu et al., 2012; Zhang et al., 2016) which has been the subject of in depth multi-omic study (Zhu et al., 2012). We checked that *R. toruloides* CBS 14 could grow in induction and repression conditions for each candidate promoter. Growth was observed in all media except where galactose was the sole carbon source; as a result *GAL1* and *GAL7* were excluded from further analysis.

**Table 2:** *R. toruloides* candidate inducible promoters.

Gene^1^	Predicted protein	Induced by	Repressed by	Reference
*GAL1*	Galactokinase	+ Galactose	+ Glucose	Ruff et al., 2009
		- Glucose		
*GAL7*	Galactose-1-phosphate uridyl transferase	+ Galactose	+ Glucose	
		- Glucose		
*SGA1*	Glucoamylase	+ Maltose	+ Xylose	Siedenberg et al., 1999
		+ Starch	+ Glucose	*Aspergillus niger GlaA*
		- Glucose		
*ICL1*	Isocitrate lyase 1	+ Acetate	+ Glucose	Barth, 1985
		- Glucose		
*ICL2*	Isocitrate lyase 2	+ Acetate	+ Glucose	
		- Glucose		
*NAR1*	Nitrate reductase	+ Nitrate	+ Ammonium	Banks et al., 1993
		- Ammonium		
*THI5*	4-amino-5-hydroxymethyl-2-methylpyrimidine phosphate synthase	- Thiamine	+ Thiamine	Maundrell, 1990
				*Schizosaccharomyces pombe nmt1*
*THI4*	Thiamine thiazole synthase	- Thiamine	+ Thiamine	Manetti et al., 1994
				*S. pombe nmt2*
*MET16*	3′ phosphoadenylsulfate reductase	- Methionine	+ Methionine	Solow et al., 2005
*CCC2*	Copper eﬄux pump	+ Copper	- Copper	Gebhart et al., 2006
				*Histoplasma capsulatum CRP1*
*CTR3*	High affinity copper transporter	- Copper	+ Copper	Labbé and Thiele, 1999
*CTR31*	Copper transporter	- Copper	+ Copper	Paralog of *CTR3*

*^1^Gene names reflect *S. cerevisiae* ortholog.*

### In-Yeast Assembly for Construction of Vectors

The high GC content of *R. toruloides* DNA, or of genes codon optimized for use in *R. toruloides*, reduces the efficiency of *in vitro* cloning techniques for assembly of plasmids for manipulation of this organism. *S. cerevisiae* in-yeast assembly is robust to the assembly of large or GC-rich fragments (Agarwal et al., 1970; Noskov et al., 2012) therefore this technique was used for vector construction. In-yeast assembly exploits the high efficiency of homologous recombination in *S. cerevisiae* to assemble multiple DNA fragments into a circular replicating plasmid (Noskov et al., 2012). The range of selectable markers is limited for *R. toruloides* so we developed G418 selection for transformation of *R. toruloides*. A cassette consisting of codon-optimized APH(3′) G418 resistance gene under regulation of the *R. toruloides GPD1* constitutive promoter was found to confer resistance to this antibiotic at a concentration of 150 μg mL^-1^. This G418 resistance marker expands the number of selection markers available for use in *R. toruloides* and uses a cheaper and safer antibiotic compared with the previously used hygromycin, bleomycin, and nourseothricin (Liu et al., 2013; Lin et al., 2014).

**FIGURE 1:**
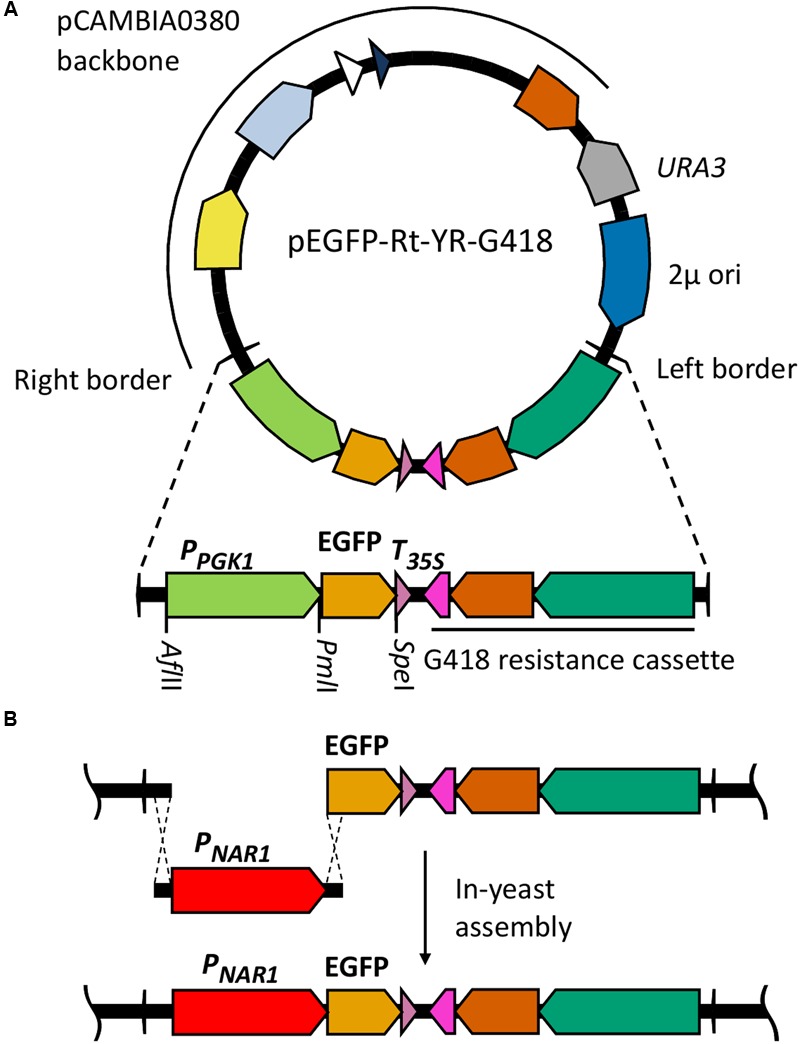
**Cloning strategy for identification of inducible promoters in *R. toruloides*. (A)** *R. toruloides* transformation vector pEGFP-Rt-YR-G418. The vector incorporates a *URA3* marker and 2 μ origin for maintenance in *S. cerevisiae*. The T-DNA region to be integrated into the *R. toruloides* genome is shown expanded; the *R. toruloides* G418 resistance cassette consists of a codon-optimized APH(3′) gene (orange) under regulation of the *R. toruloides GPD1* promoter (green) and *N. crassa* beta tubulin terminator (pink). The pCAMBIA0380 backbone contains, in a clockwise direction: the right border sequence (RB); pVS1 *StaA* stability region (yellow) and *RepA* replication origin (light blue) for maintenance in *A. tumefaciens*; pBR322 *bom* (white) and *ori* (dark blue) for maintenance in *E. coli*; *kanMX* kanamycin resistance cassette (orange). pCAMBIA0380 also provides the left border sequence (LB). **(B)** Cloning strategy for inserting promoters of interest upstream of EGFP gene. The promoter of interest, the *NAR1* promoter in the example shown, is amplified with 25-bp overhangs complementary to regions flanking the insertion site. This is co-transformed into *S. cerevisiae* along with pEGFP-Rt-YR-G418 pre-digested with *Afl*II and *Pml*I. *In vivo* homologous recombination inserts the promoter upstream of the EGFP gene in the vector.

Plasmid pC-G418-YR (Kilaru and Steinberg, 2015) *Zymoseptoria tritici* transformation vector was used as a base for assembly of *R. toruloides* transformation vectors. This plasmid is a derivative of pCAMBIA0380 modified to include a *URA3* selection marker and a 2 μ origin of replication in the vector backbone, facilitating maintenance in *S. cerevisiae*. It also includes a G418 resistance marker regulated by *Z. tritici* α-tubulin promoter and *Neurospora crassa* β-tubulin terminator in the T-DNA region. We excised the *Z. tritici* promoter and G418 resistance marker and replaced them with the *R. toruloides* codon-optimized G418 resistance marker under regulation of *R. toruloides* CBS 14 *GPD1* constitutive promoter (Liu et al., 2013). We also inserted a synthetic construct into the T-DNA region containing a codon-optimized EGFP gene under regulation of the *R. toruloides PGK1* promoter (Lin et al., 2014; mutated to include a *Pml*I cut site at the -7 to -12 position) and the CMV35S terminator. An *Afl*II cut site was incorporated upstream of the promoter and a *Spe*I site immediately downstream of the EGFP. The resulting plasmid, pEGFP-Rt-YR-G418 (**Figure 1A**), is designed such that the *PGK1* promoter or the EGFP gene can easily be exchanged by digestion with *Afl*II/*Pml*I or *Pml*I/*Spe*I, respectively, and the promoter or gene of interest inserted by in-yeast assembly (**Figure 1B**). A second variant (pEGFP-Rt-YR-Hyg) was produced replacing the G418 resistance gene with a codon-optimized hygromycin resistance marker (Liu et al., 2013).

To test each of the 10 promoters, 1500 bp upstream of the translational start site was amplified by PCR and inserted in place of the *PGK1* promoter, upstream of the EGFP reporter gene (**Figure 1B**).

### GFP Screening Identifies *NAR1, ICL1, CTR3* and *MET16* Inducible Promoters in *R. toruloides*

Each promoter-EGFP construct was transformed into *R. toruloides* haploid strain CBS 14. To identify which candidate promoters can be used as regulatable promoters, cultures were grown for 16 h under induced and repressed conditions and EGFP fluorescence measured by flow cytometry. To minimize any positional effects from the locus of integration of the T-DNA into the *R. toruloides* genome, each test was performed on three independently transformed biological replicates.

**FIGURE 2:**
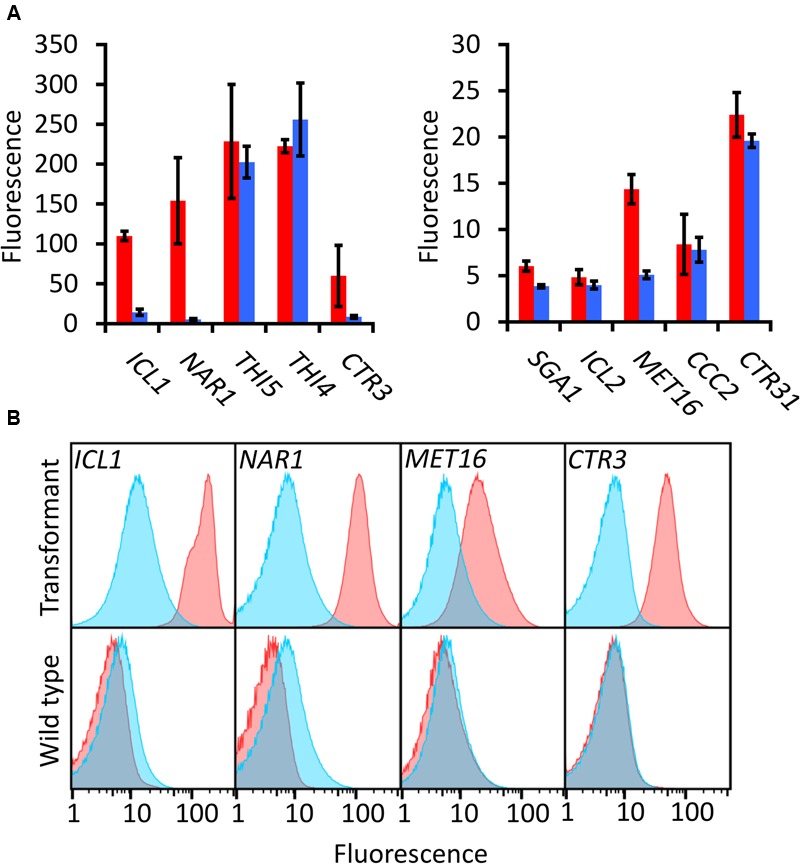
**EGFP-based screening of ten candidate inducible promoters in *R. toruloides*. (A)** Fluorescence of *R. toruloides* cells with EGFP regulated by test promoters after overnight growth in induced conditions (red bars) and repressed conditions (blue bars). Fluorescence was measured by flow cytometry and the median cellular fluorescence determined for ≥500 000 cells per culture. Bars indicate the mean of three independently transformed biological replicates with the standard deviation shown as error bars. Induction and repression mean values are significantly different (*p* < 0.05) for promoters *ICL1, NAR1, SGA1* and *MET16*, as determined by student’s *t*-test. **(B)** Representative histograms showing fluorescence of cells in induced (red) and repressed (blue) conditions for the *ICL1, NAR1, MET16* and *CTR3* promoters. Upper panels show transformant cells with EGFP under the regulation of each indicated promoter; lower panels show autofluorescence of untransformed cells under growth conditions identical to the transformants above.

Of the candidates screened, the promoters of *ICL1, NAR1*, and *MET16* demonstrated inducibility (**Figure 2A**). The *NAR1* promoter displayed high levels of induced expression surpassed only by the *THI5* and *THI4* constitutive promoters. This promoter also exhibited low expression when repressed (measured induction ratio = 29).

The *ICL1* promoter also displayed high levels of induced expression; however, *ICL1* repression was incomplete in the presence of glucose (measured induction ratio = 7.6). This is consistent with activity observed in the oleaginous ascomycete yeast *Yarrowia lipolytica* as well as the economically important *Komagataella* (formerly *Pichia*) *pastoris* (Barth, 1985; Menendez et al., 2003). Acetic acid has been proposed as a feedstock for industrial growth of *R. toruloides* due to its low cost (Huang et al., 2016), and under these conditions the *ICL1* promoter would be induced. Such a system has been proposed for protein production in *K. pastoris*, as an alternative to the commonly used methanol-induced *AOX* promoter (Menendez et al., 2003).

The *MET16* promoter had a low induced expression level (about one tenth the strength of the induced *NAR1* promoter) and also a low induction ratio. However, under repressed conditions the measured fluorescence was comparable to the autofluorescence of untransformed cells under identical conditions (**Figure 2B**), therefore the apparent induction ratio of 2.8 should be considered a minimum.

The *CTR3* promoter exhibited strong repression in the presence of copper and had a medium level of induction in its absence; however, there was a large degree of variation between the replicates. For this reason the copper chelator BCS was added to induction medium in all subsequent experiments; this resulted in consistent and significant induction of the *CTR3* promoter. The *NAR1* and *ICL1* promoters require changes in nitrogen or carbon sources, respectively, between induced and repressed conditions; this would have effects on global metabolism whereas the copper starvation conditions for induction of the *CTR3* promoter are unlikely to lead to such gross changes in metabolism (Ouyang et al., 2015). The *CTR3* inducible promoter can therefore be useful where background metabolic considerations are important, such as in a laboratory setting.

Other promoters screened either showed constitutive activity (*THI5, THI4, CTR31*) or little to no induced fluorescence under the conditions tested (*SGA1, ICL2* and *CCC2*).

### Gene Expression Is Activated within 4–16 h of Promoter Induction

**FIGURE 3:**
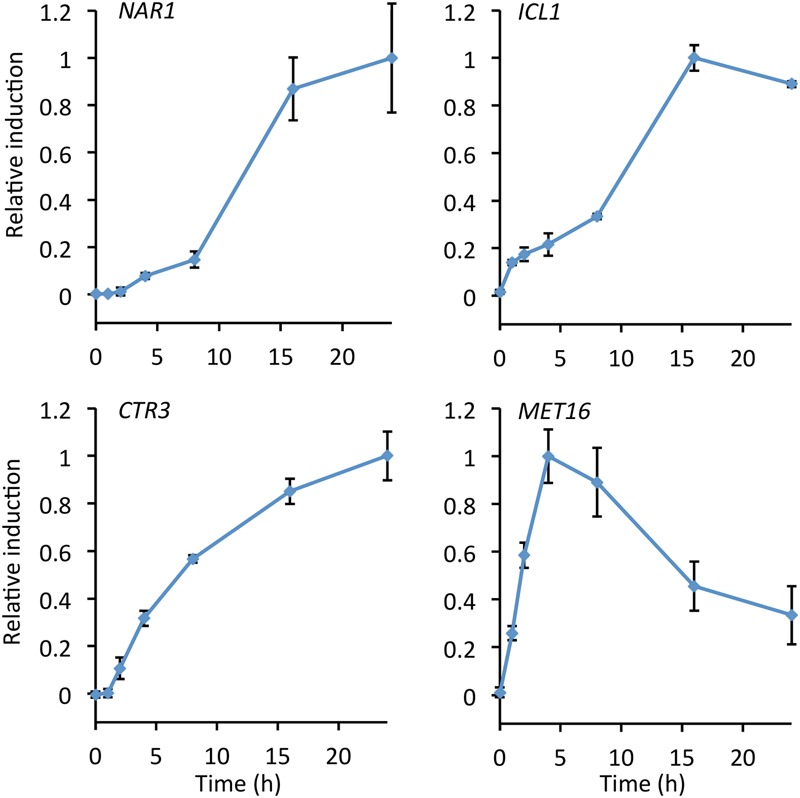
**Time course showing relative promoter induction up to 24 h after transfer to inducing conditions.** Cultures were grown in repressive medium overnight before cultures were washed, split, and transferred to fresh induction/repression medium. Samples were taken at the times indicated and fluorescence measured. Induction was calculated as fluorescence under induced conditions minus fluorescence under repressed conditions and normalized to maximum observed induction. Points show the mean of three independently transformed biological replicates; error bars indicate standard deviation.

The rate of induction for each of the four promoters was measured by performing a time course over 24 h from transfer to induction medium, after overnight culture in repression medium (**Figure 3**). Autofluorescence due to carotenoids produced during late log and stationary phase gives high background after 24 h making measurements unreliable (Kleinegris et al., 2010; Lee et al., 2014).

The *MET16* promoter was the fastest to induce, reaching a maximum after 4 h and declining after 8 h. This promoter may therefore be suitable for experiments where rapid induction is desirable but high-level expression is not required. Both the *NAR1* and *ICL1* promoters showed greatest increases in expression after 8 h, reaching maxima at around 16 h. In the presence of the copper chelator BCS, induction of the *CTR3* promoter started at 2 h and increased asymptotically up to 24 h (**Figure 3**).

Cultures with sodium acetate as the sole carbon source grew slowly relative to cultures with glucose. In a laboratory setting this may be problematic when comparing the biology of cultures in induced and repressed conditions for the *ICL1* promoter, and in an industrial setting may cause reduction in yield; however, this could be overcome by using a two-stage fermentation, initially growing with glucose and then switching to growth on acetate.

### Conditional Mutant Rescue Using the *NAR1* Promoter

To investigate controllable mutant rescue the *R. toruloides leu2* mutant strain NCYC 1585 was used (Tully, 1985; Lin et al., 2012). The EGFP gene in vector pEGFP-Rt-YR-G418 was replaced by *LEU2* from *R. toruloides* CBS 14 to give plasmid pLeu-Rt-YR-G418. This construct rescued *R. toruloides* NCYC 1585 growth on leucine deficient medium; transformants could be selected either by growth on leucine-minus medium or by G418 resistance.

**FIGURE 4:**
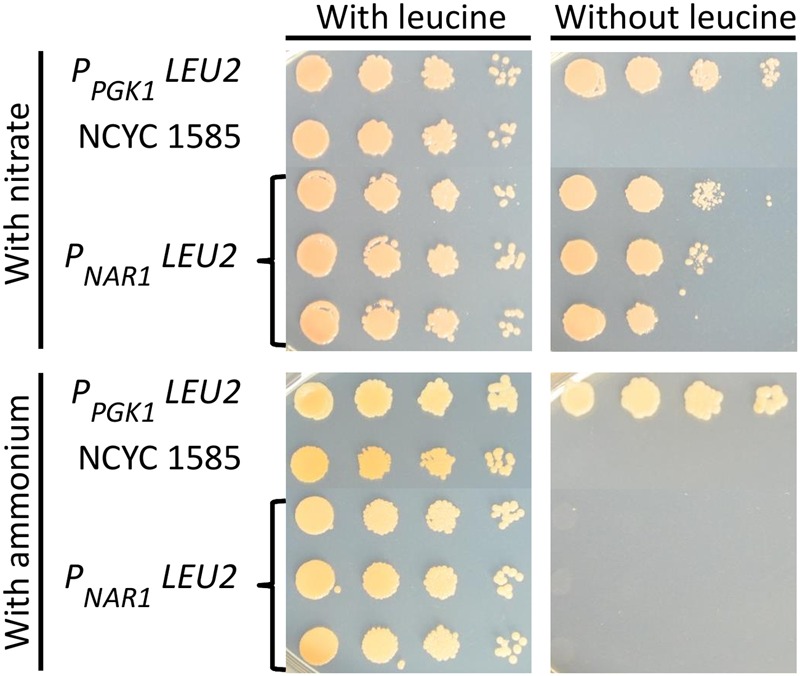
**Conditional rescue of *leu2* mutant *R. toruloides* strain NCYC 1585 with *LEU2* under regulation of the *NAR1* promoter.** Cells from three independent transformant lines were grown overnight in induction medium with leucine (100 mg L^-1^) and plated on to YNB with 2% agarose with either 3.5 g L^-1^ ammonium sulfate or 0.78 g L^-1^ potassium nitrate and allowed to grow for 4 days. Cells transformed with *LEU2* under the regulation of the constitutive *PGK1* promoter and untransformed NCYC 1585 cells were included as positive and negative controls, respectively.

The promoter driving the *LEU2* gene was then exchanged for each of the four inducible promoters and these constructs transformed into *R. toruloides* NCYC 1585, selecting for transformants with G418. Transformant strains were grown overnight in induction media supplemented with leucine and spot plated to solid induction/repression media with or without leucine. All transformants were able to grow under induction conditions in the absence of leucine, indicating mutant rescue by *LEU2* under the transcriptional control of each of the four inducible promoters. On solid medium under repressive conditions, transformants carrying *LEU2* under the regulation of the *NAR1* promoter were unable to grow (**Figure 4**) demonstrating conditional rescue of *leu2 R. toruloides* using the *NAR1* promoter, and confirming low expression levels under repressive conditions for this promoter.

Cells transformed with *LEU2* under regulation of *ICL1, CTR3*, or *MET16* promoters were able to grow under repressive conditions indicating incomplete repression (Supplementary Figure 1). This could reflect strain differences, as the NCYC 1585 *leu2* strain is a derivative of *R. toruloides* strain CBS 349 which shares only 87 % DNA sequence identity with CBS 14 (Kumar et al., 2012; Zhang et al., 2016), although the two strains can mate (Banno, 1967). Alternative explanations are possible, for example regulatory elements within a *LEU2* intron enhancing promoter expression, as in the case of the *DAO1* promoter in strain CBS 349 (Liu et al., 2015), but this would require further study to explore.

### Functional Dissection of *R. toruloides* Inducible Promoters

**FIGURE 5:**
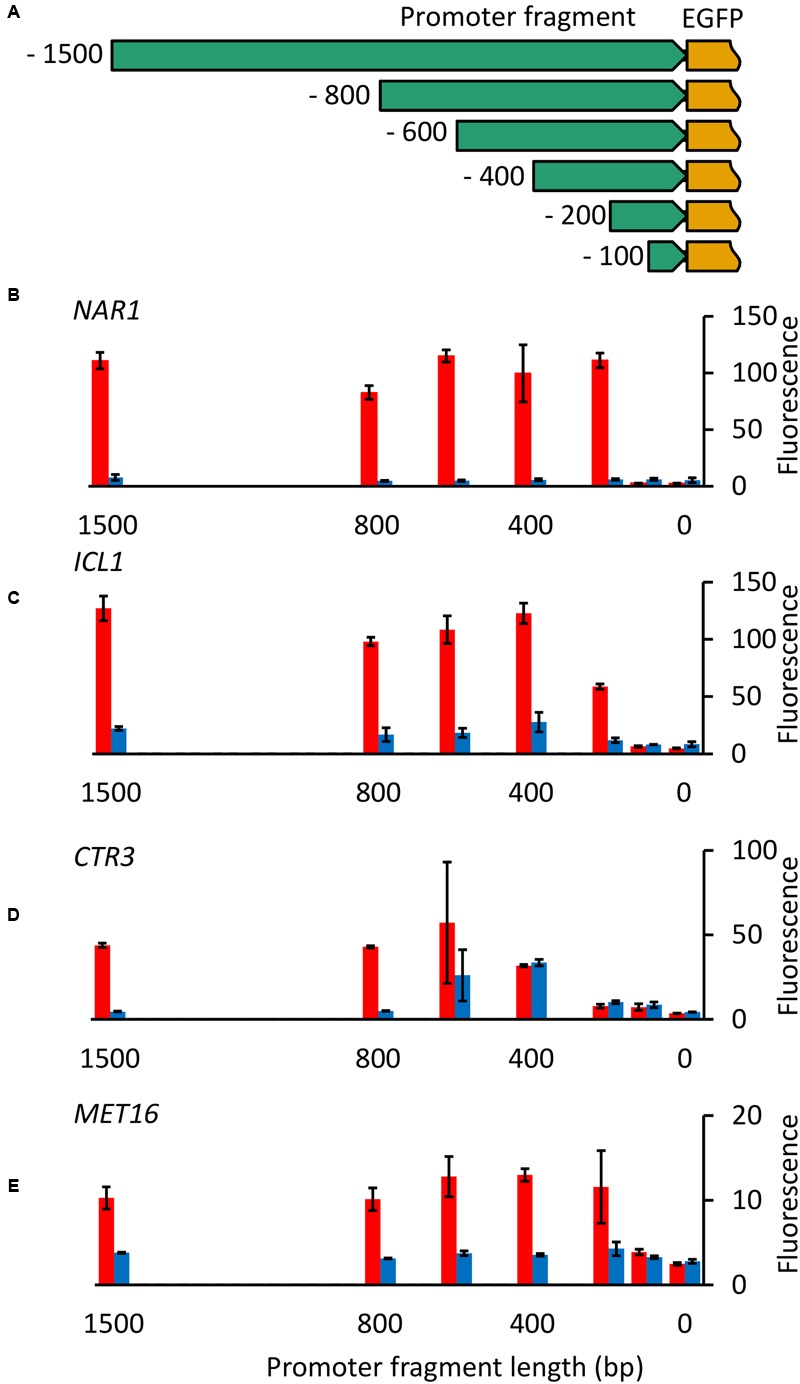
**Nested deletion of promoter fragments. (A)** Diagram of constructs produced. For each of the four inducible promoters fragments 100, 200, 400, 600, 800, and 1500 bp in length upstream from the ATG were cloned upstream of the codon-optimized EGFP gene. **(B–E)** EGFP fluorescence of cells carrying truncated versions of *NAR1* **(B)**, *ICL1* **(C)**, *CTR3* **(D)** and *MET16* **(E)** promoters under induced (red) and repressed (blue) conditions. Cells were grown overnight and then transferred to fresh induction/repression media. Fluorescence was measured after 16 h growth in induction/repression media (8 h for *MET16*). Bars show the mean fluorescence of three independently transformed biological replicates; error bars indicate standard deviation. Induction and repression mean values are significantly different (*p* < 0.05) for the following promoter fragment lengths: *NAR1* ≥ 200 bp; ICL1 ≥ 200 bp; *CTR3* ≥ 800 bp; *MET16* ≥ 400 bp and 100 bp.

Initially promoter fragments tested were all 1500 bp in length. To identify the minimum size of each promoter required for controllable gene expression and the location of regulatory elements, nested deletions of each of the four inducible promoters were cloned upstream of the EGFP gene (**Figure 5A**) and fluorescence measured for *R. toruloides* CBS 14 transformants under induced and repressed conditions.

With the *NAR1* promoter no activity was observed with the 100-bp fragment but full regulation was observed with fragments 200 bp and longer (**Figure 5B**), demonstrating all necessary controlling elements are present in this short region. Similarly, for the *ICL1* and *MET16* promoters, little or no activity was observed with the 100-bp fragments, full regulation required 400-bp fragments, with 200 bp giving partial activity under induced conditions for *ICL1* (**Figures 5C,E**). *CTR3* promoter cut-downs showed a more interesting pattern: 100- and 200-bp fragments showed little activity, the 400-bp fragment was constitutively active, and the 800-bp and 1500-bp fragments exhibited full regulation (**Figure 5D**).

To identify functional elements within essential promoter regions, a motif search was performed using MEME for conserved elements between orthologous promoters in *R. toruloides* and related members of the Pucciniomycotina. In both the *ICL1* and *CTR3* promoters, CT-rich boxes were identified in the -50 to -40 region relative to the start codon. Similar elements have been observed in the *R. toruloides GPD1* and *DAO1* promoters (Liu et al., 2013, 2015) indicating this is a highly conserved element in *R. toruloides*. Such an element has also been observed in other filamentous fungi where it is proposed to be responsible for targeting the translational start site (Punt et al., 1990).

In the *CTR3* promoter a second conserved box was identified at -583 to -602 with the consensus GCRAAAANNGCGCATC. The 400-bp promoter fragment showed constitutive induction, the 600-bp fragment exhibited variable repression and the 800-bp fragment full repression in the presence of copper; this sequence element could therefore be responsible for repression of this promoter in the presence of copper. Other instances of this element were identified in *R. toruloides* promoters using FIMO (Grant et al., 2011) and the genes adjacent to the top 10 hits identified. Apart from *CTR3*, the top hit was upstream of a vacuolar ABC heavy metal transporter, a gene likely to be regulated by copper, and the second hit was in the promoter for salicylate hydroxylase, the product of which (catechol) is toxic in the presence of heavy metals (Schweigert et al., 2001) and thus would likely be repressed in the presence of copper. The motif was also identified 283 bp downstream of a second gene annotated as a copper transporter. Given the range at which this element acts it is possible that this element can act on the promoter of this gene from this location.

## Conclusion

We have characterized four inducible promoters to allow controllable expression in the oleaginous yeast *R. toruloides*, designed vectors for efficient cloning of its high-GC DNA, and added to the range of useful selectable markers for this yeast. The *NAR1* promoter is strongest when induced, shows tight regulation under repressed conditions in two *R. toruloides* strain backgrounds, has a short 200 bp functional sequence, and would be the first choice promoter in many cases. However, each promoter has its own individual characteristics that render it suitable for particular applications, and together they provide a suite of complementary regulatory elements for controlling gene expression in this yeast.

## Author Contributions

Experiments were conceived and designed by AJ and SA with assistance from JL. Experiments were performed by AJ. Analysis was performed by AJ and SA with assistance from JL. SA and JL supervised the project. The manuscript was written by AJ and SA with assistance from JL.

## Conflict of Interest Statement

The authors declare that the research was conducted in the absence of any commercial or financial relationships that could be construed as a potential conflict of interest.
