# Supplementary material for: Four Inducible Promoters for Controlled Gene Expression in the Oleaginous Yeast Rhodotorula toruloides
Source: Front Microbiol. 2016 Oct 21;7:1666. doi: 10.3389/fmicb.2016.01666 (PMC5073140; doi:10.3389/fmicb.2016.01666)
Supplement: Supplementary file 1 [file Image_1.PDF]

Supplementary Material

Four inducible promoters for controlled gene expression in the oleaginous yeast *Rhodotorula toruloides*

Alexander M. B. Johns, John Love, Stephen J. Aves*

*** Correspondence:** Stephen J. Aves: S.J.Aves@ex.ac.uk

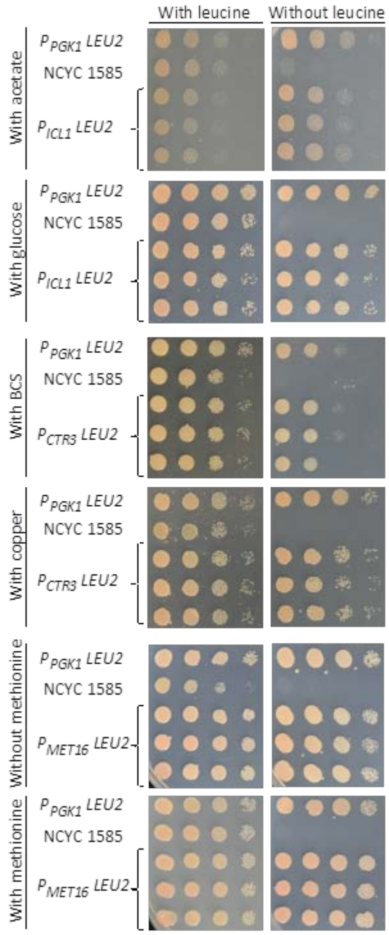

Supplementary Figure 1. Rescue of *leu2 R. toruloides* strain NCYC 1585 with *LEU2* under regulation of the *ICL1*, *CTR3* and *MET16* promoters. In each case three independent transformant lines were grown overnight in induction media with leucine (100 mg L^{-1}) and plated on to solid YNB with 20 g L^{-1} glucose modified for promoter induction or repression, with or without leucine and grown for two days. For induction of P_{ICL1} glucose was replaced with 200 mM sodium acetate; for induction of P_{CTR3}, media were without copper and supplemented with 100 µM bathocuproinedisulfonic acid (BCS), for repression 20 µM $CuSO_4$ was added; for repression of P_{MET16} 1 mM methionine was added. *R. toruloides* NCYC 1585 with *LEU2* under regulation of the constitutive *PGK1* promoter and untransformed *R. toruloides* NCYC 1585 were included as positive and negative controls respectively.
